# IL22/IL-22R Pathway Induces Cell Survival in Human Glioblastoma Cells

**DOI:** 10.1371/journal.pone.0119872

**Published:** 2015-03-20

**Authors:** Hussein Akil, Amazigh Abbaci, Fabrice Lalloué, Barbara Bessette, Léa M. M. Costes, Linda Domballe, Sandrine Charreau, Karline Guilloteau, Lucie Karayan-Tapon, François-Xavier Bernard, Franck Morel, Marie-Odile Jauberteau, Jean-Claude Lecron

**Affiliations:** 1 Laboratoire Homéostasie Cellulaire et Pathologies (LHCP-EA 3842), Faculté de Médecine et de Pharmacie, Université de Limoges, Limoges, France; 2 Laboratoire Inflammation, Tissus Epithéliaux et Cytokines (LITEC-EA 4331), Université de Poitiers, Poitiers, France; 3 INSERM U1084, Université de Poitiers, Poitiers, France; 4 Laboratoire de Cancérologie Biologique, CHU de Poitiers, Poitiers, France; 5 BIOalternatives, Gençay, France; 6 Service Immunologie et inflammation, CHU de Poitiers, Poitiers, France; French National Centre for Scientific Research, FRANCE

## Abstract

Interleukin-22 (IL-22) is a member of the IL-10 cytokine family that binds to a heterodimeric receptor consisting of IL-22 receptor 1 (IL-22R1) and IL-10R2. IL-22R expression was initially characterized on epithelial cells, and plays an essential role in a number of inflammatory diseases. Recently, a functional receptor was detected on cancer cells such as hepatocarcinoma and lung carcinoma, but its presence was not reported in glioblastoma (GBM). Two GBM cell lines and 10 primary cell lines established from patients undergoing surgery for malignant GBM were used to investigate the expression of IL-22 and IL-22R by using quantitative RT-PCR, western blotting and confocal microscopy studies. The role of IL-22 in proliferation and survival of GBM cell lines was investigated *in vitro* by BrdU and ELISA cell death assays. We report herein that the two subunits of the IL-22R complex are expressed on human GBM cells. Their activation, depending on exogenous IL-22, induced antiapoptotic effect and cell proliferation. IL-22 treatment of GBM cells resulted in increased levels of phosphorylated Akt, STAT3 signaling protein and its downstream antiapoptotic protein Bcl-xL and decreased level of phosphorylated ERK1/2. In addition, IL-22R subunits were expressed in all the 10 tested primary cell lines established from GBM tumors. Our results showed that IL-22R is expressed on GBM established and primary cell lines. Depending on STAT3, ERK1/2 and PI3K/Akt pathways, IL-22 induced GBM cell survival. These data are consistent with a potential role of IL-22R in tumorigenesis of GBM. Since endogenous IL-22 was not detected in all studied GBM cells, we hypothesize that IL-22R could be activated by immune microenvironmental IL-22 producing cells.

## Introduction

Interleukin 22 (IL-22), a member of the IL-10 cytokine family, is produced by several subsets of lymphocytes such as CD4^+^ T helper 17 (Th17) cells (able to produce also IL-17A and IL-17F) and Th22 cells, CD8^+^ cytotoxic T cells, natural killer (NK) cells, γδ T cells and lymphoid tissue inducer (LTi)-like cells [1]. IL-22 signals through a heterodimeric receptor composed of two subunits, the specific receptor IL-22R1 and the shared subunit, IL-10R2 [2, 3]. Unlike IL-10 and most of the cytokines, IL-22 has no effect on immune cells [4, 5].

In agreement, IL-22R1 is not expressed on immune cells [6] but selectively detected on epithelial cells, *ie* keratinocytes [7], hepatocytes [8], pancreatic cells [9], lung cells [10], kidney cells [11] and colonic epithelial cells [12]. Binding of IL-22 to its receptor activates the Janus kinase 1 (JAK1), followed by the signal transducers and activators of transcription protein 3 (STAT3) and STAT5 pathways [13, 14]. IL-22 also activates the MAP kinase pathways such as the extracellular signal regulated kinase 1/2 (ERK1/2), mitogen activated protein kinases (MAPK) like c-Jun N-terminal kinase (JNK) and p38 [1, 8, 13]. In addition, IL-22 activates the phosphatidylinositide 3-Kinase-Akt-mammalian target of rapamycin (PI3K-Akt-mTOR) pathway [8, 15, 16].

The biological role of IL-22 was initially described in hepatoma [5], pancreatic acinar [9] cells and keratinocytes [7], thereafter reported to be involved in the pathogenesis of numerous inflammatory diseases, notably in skin inflammation such as psoriasis [17, 18]. Indeed, IL-22 induces an inflammatory phenotype on keratinocytes and inhibits their differentiation [7, 19]. Beside these well characterized immunopathological functions on epithelial tissues, the role of IL-22 in cancer cell biology has been recently reported in lung [20], gastric [21], colorectal [22, 23], pancreatic [24, 25], and hepatocellular carcinomas [26], whose cells expressed the IL-22R1/IL-10R2 receptor subunits. Indeed, IL-22 was described as an autocrine factor of human lung cancer cells contributing to cancer cell survival and resistance to chemotherapy, and its therapeutic effect was showed in an *in vivo* xenograft model using IL-22-RNAi plasmids [20]. In hepatocellular carcinoma, tumor infiltrated leukocytes were significantly enriched in IL-22 expressing cells. Moreover, IL-22 expression was positively correlated with tumor growth, metastasis and tumor stages [26]. *In vitro*, IL-22 induced hepatocyte cells survival and proliferation by activating STAT3 phosphorylation and Akt [13]. The role of IL-22 in colorectal cancer was also investigated by Jiang et al., who demonstrated that upregulation of IL-22 in human colon cancer microenvironment enhances tumor growth, inhibition of apoptosis and promotion of metastasis by activating STAT3 phosphorylation [22]. In addition, IL-22 levels were positively correlated with colorectal cancer tumor stages [27], and serum IL-22 level was elevated in chemoresistant patients [23]. In pancreatic cancer, IL-22 enhances metastatic ability of pancreatic ductal adenocarcinoma cell lines, and high expression of IL-22 and IL-22R1 was associated to poor prognosis [25].

In the central nervous system, the presence of human Th17 lymphocytes and their deleterious role were described in multiple sclerosis lesions. Kebir and colleagues reported the expression of IL-17 and IL-22 receptors on blood-brain barrier endothelial cells during multiple sclerosis lesions and in experimental autoimmune encephalomyelitis (EAE), a mouse model of multiple sclerosis [28]. They showed that Th17 lymphocytes transmigrate efficiently from the systemic compartment into the central nervous system across the human blood-brain barrier endothelial cells. In addition, the presence of Th17 lymphocytes has been recently reported in primary human malignant GBM [29]. These authors showed that IL-17A mRNA levels in human GBM were higher than in normal human brain. To the best of our knowledge, the expression of IL-22R in GBM has never been reported. Although the mechanisms underlying the malignant transformation of glial cell into GBM are not well elucidated, the hypothesis of an early block in the differentiation of glial progenitors into differentiated cells is issued [30–32].

Given the implication of Th17 lymphocytes in multiple sclerosis lesions and their presence in human GBM, the target tissues of Th17 cell-derived IL-22, its ability to inhibit the cellular differentiation and the association of its expression to various cancer, we questioned on a direct role of IL-22 on GBM cells. Herein, we report that human GBM cell lines as well as GBM-initiating cells express the two subunits of the functional IL-22 receptor. Activation of IL-22 receptor by IL-22 induces GBM cell survival and proliferation by activating STAT3 and PI3k/Akt pathways. Collectively, a role for IL-22 in the growth and potential invasiveness of GBM is suggested.

## Materials and Methods

### Cell lines and cell culture

Human GBM cell lines were purchased from the American Type Culture Collection (ATCC/LGC Standards). U87MG cells were maintained in MEM medium with Earle’s salts supplemented with 10% heat-inactivated Fetal Calf Serum (FCS), 1mM sodium pyruvate, 1% non essential amino acids, 50 IU/mL penicillin and 50 mg/mL streptomycin. U118MG cells were cultured in DMEM, high glucose, glutamax medium supplemented with 10% FCS, 1mM sodium pyruvate, 50 IU/mL penicillin and 50mg/mL streptomycin (all from Gibco/Life Technologies), at 37°C under humidified atmosphere and 5% CO2. NHEK were obtained from surgical samples of healthy breast skin and cultured as previously described [7]. Cells were serum-starved for 4 hours (h) before recombinant IL-22 treatment. The recombinant human IL-22 (20 ng/mL) was purchased from R&D Systems, recombinant human SuperFasLigand (100 ng/mL) from Enzo Life Sciences, LY294002 and U0126 from Cell Signaling Technology. Vehicle (0.05% v/v DMSO) was used as control.

### GBM-initiating cells culture

Tumor samples were obtained within 30 min after surgical resection from ten adult GBM patients (Department of Neurological Surgery, Poitiers University Hospital, France). Written informed consent forms were obtained from all patients enrolled in this study, which was approved by the Poitiers University Hospital Ethics Committee (DHOS/OPRC/FCnotif-tumoro-jun04: 04056) and in accordance with the Declaration of Helsinki. Tumor tissues were washed and mechanically dissociated. Cells were resuspended in a defined serum free medium and cultured as previously described [33].

### Real-time RT-PCR analysis

Total cellular RNA was isolated using Trizol reagent (Invitrogen/Life Technologies) and treated with DNase I (0.05 U/μl; Clontech/Ozyme). Four μg of total RNA were reverse transcribed using Superscript II Reverse Transcriptase (Invitrogen) according to the manufacturer’s instructions. Quantitative PCR was carried out using the LightCycler-FastStart DNA Master^PLUS^ SYBR Green I kit (Roche Molecular Diagnostic). The reaction components were 1X FastStart DNA Master SYBR Green I, 3 mM MgCl2, and 0.5 μM of forward and reverse primers as previously described [18, 19]. A calibration curve was performed with purified PCR products of target genes. The cycling conditions comprised 10 min polymerase activation at 95°C and 45 cycles at 95°C for 10 s, 64°C for 5 s, and 72°C for 18 s with a single fluorescence measurement. Melting curve analysis, obtained by increasing the temperature from 60°C to 95°C with a heating rate of 0.1°C per second and a continuous fluorescence measurement, revealed a single, narrow peak of suspected fusion temperature. The Delta Ct mathematical model was used to determine the relative quantification of target genes compared with the GAPDH reference gene.

### Western blotting

Anti-IL-22R1 antibody (Ab) (0.5 μg/mL) was purchased from Abcam, anti-IL-10R2 Ab (1 μg/mL) from R&D Systems, anti-phospho-STAT3 (Tyr705) (1:2000), anti-STAT3 (1:1000), anti-phospho-Akt (1:2000), anti-Akt (pan) (1:2000) Abs from Cell Signaling Technology and anti-Actin Ab (1:10000) from Sigma-Aldrich. Cell lines cultures were lysed using 1X Cell lysis buffer (Cell Signaling Technology), according to the manufacturer’s instructions. Protein concentration was determined using Bradford protein concentration assay (Sigma). SDS-PAGE was performed and the proteins were electro-blotted onto Immobilon PVDF membranes (Merck Millipore). After 1 h of incubation at room temperature in blocking solution (5% non-fat dry milk in 1X TBS-0.1% Tween-20), the membranes were exposed to the specific primary Abs in blocking solution overnight at 4°C. Then, membranes were washed thrice for 5 min with TBS-0.1% Tween-20 and the immunoreactions were detected by horseradish peroxidase-conjugated secondary Ab to mouse or rabbit Ig (Dako) diluted at 1:2000 in blocking solution for 1 h at room temperature. After washing, visualization of immunocomplexes was accomplished using the Immobilon Western Chemiluminescent HRP Substrate (Merck Millipore). Protein-loading control was performed with anti-Actin Ab. Western blots were scanned using a bio-imaging system (Genesnap; Genetool; Syngene). Densitometric analyses were performed using an IMAGEJ software program (National Institutes of Health, Bethesda, MD, USA http://rsb.info.nih.gov/ij/). Protein expression was determined in relative units in reference to actin expression.

### Immunofluorescence

Cells grown on 14-mm coverslips (Fisher Scientific) were fixed with 4% formaldehyde in 1X PBS at room temperature for 15 min (in fume hood), rinsed three times in PBS and permeabilized or not with ice-cold 100% methanol for 10 min at -20°C. After 5 min of wash in PBS, nonspecific binding was blocked by 30 min incubation with blocking buffer (1X PBS-5% normal goat serum with or without 0.3% Triton X-100) at room temperature. Coverslips were then incubated overnight at 4°C in Ab dilution buffer (1X PBS-1% BSA with or without 0.3% Triton X-100) with the primary Ab. The following primary Abs were used: rabbit polyclonal anti-IL-22R1 Ab (10 μg/mL; Abcam), mouse monoclonal anti-IL-10R2 Ab (5 μg/mL; R&D Systems), rabbit monoclonal anti-phospho-STAT3 (Tyr705) (1:100; Cell Signaling Technology) and mouse monoclonal anti-STAT3 Ab (1:1600; Cell Signaling Technology). Cells were washed 3 times in PBS, and incubated for 2 h at room temperature (in dark) with 488 nm Alexa Fluor-conjugated secondary Abs (Invitrogen) diluted 1:1000 in Ab dilution buffer. After 3 washes in PBS, nuclei were stained for 5 min with DAPI (Sigma). After intensive washes, coverslips were inverted on slides and mounted with Dako Fluorescent Mounting Medium. Isotype controls were cells incubated with irrelevant normal rabbit or mouse IgG (Sigma). Pictures were taken using a confocal microscope (Carl Zeiss, LSM 510).

### ELISA

IL-22 in U87MG and U118MG cell line supernatants was detected by sandwich ELISA (Peprotech), according to manufacturer’s instructions. Concentrations below the detection limit (5pg/mL) were considered undetectable.

### Cell proliferation assays

Cell proliferation was measured using the BrdU cell Proliferation Assay (Cell Signaling Technology), according to the manufacturer’s instructions. Proliferation values were measured using a microplate photometer (Thermo Fisher Scientific). Absorbance values were measured at 450 nm.

### Apoptosis assay

Apoptosis was measured by the detection of cytoplasmic soluble nucleosomes using a calorimetric assay, Cell Death Detection ELISAPLUS kit (Roche) according to the manufacturer’s instructions. Absorbance values were measured at 405–490 nm dual wavelengths.

### Statistical analysis

Statistical significance was determined by a one-way analysis of variances (ANOVA) with statview 5.0 software (Abacus Concepts). *P* values < 0.05 were considered significant. Mean and SEM values were obtained from at least 3 independent experiments.

## Results

### GBM cell lines express IL-22R1 and IL-10R2 receptors but not Interleukin-22

**Fig 1:**
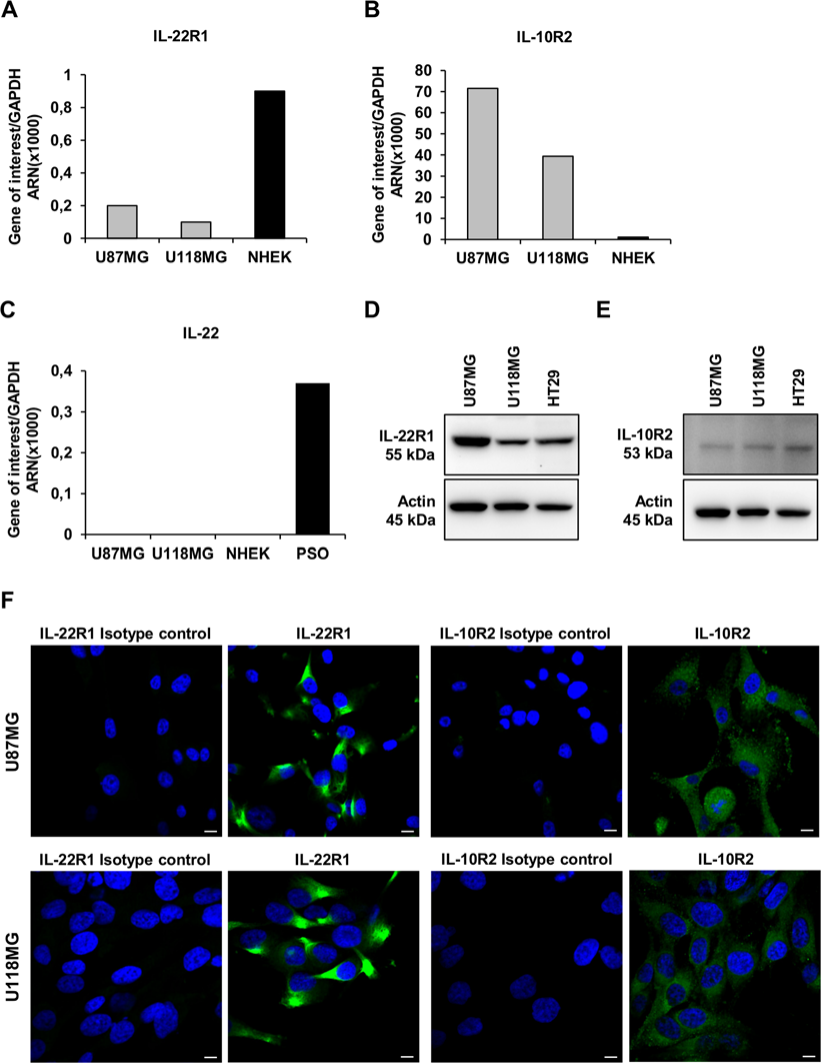
Expression of IL-22, IL-22R1 and IL-10R2 in GBM cell lines. (A-C) Quantitative RT-PCR analysis of IL-22 and its two receptors (IL-22R1, IL-10R2) in total RNA extracted from U87MG and U118MG cell lines. Positive controls were the human epidermal keratinocytes (NHEK) for IL-22R1 (A) and IL-10R2 (B) and the human psoriatic skin biopsies (PSO) for IL-22 expression (C). Target gene expression was normalized to the housekeeping GAPDH mRNA. (D, E) Detection of IL-22R1 (D) and IL-10R2 (E) proteins assessed by western blot analysis in the two studied cell lines. Positive control was the colorectal cancer cell line HT29 for both IL-22R1 and IL-10R2 expression. Actin was used as a loading protein control. (F) Confocal microscopy analysis of IL-22R1 and IL-10R2 labeled with specific antibodies and revealed with Alexa fluor-488 conjugated fluorescent antibodies (green) in U87MG and U118MG cell lines. Nuclei were counter stained with the blue-fluorescent DNA stain DAPI. Scale bars, 10μm.

The two subunits of the functional IL-22R complex, IL-22R1 and IL-10R2 were detected in the U87MG and the U118MG GBM cell lines both at mRNA (Fig. 1A and 1B) and protein (Fig. 1D and 1E) levels with a higher expression in U87MG cell line. By using NHEK as positive controls for mRNA expression, we showed lower expression levels for IL-10R2, but higher levels for IL-22R1 than in GBM cell lines. By contrast, the IL-22 cytokine transcript was not detectable in both the GBM cell lines nor NHEK, whereas it is present in the psoriatic skin samples, reported to express IL-22 mRNA [18] (Fig. 1C). In agreement, IL-22 was not detected (<5pg/mL) in culture supernatant of both GBM cell lines (data not shown). The membranous and cytoplasmic expression of IL-22R1 and IL-10R2 were detected by immunofluorescence in the two GBM cell lines (Fig. 1F), in agreement with the transcriptional and western blot studies, suggesting that GBM cancer cell lines have the ability to respond to IL-22 stimulation.

### Interleukin-22 induced GBM cell proliferation and survival

**Fig 2:**
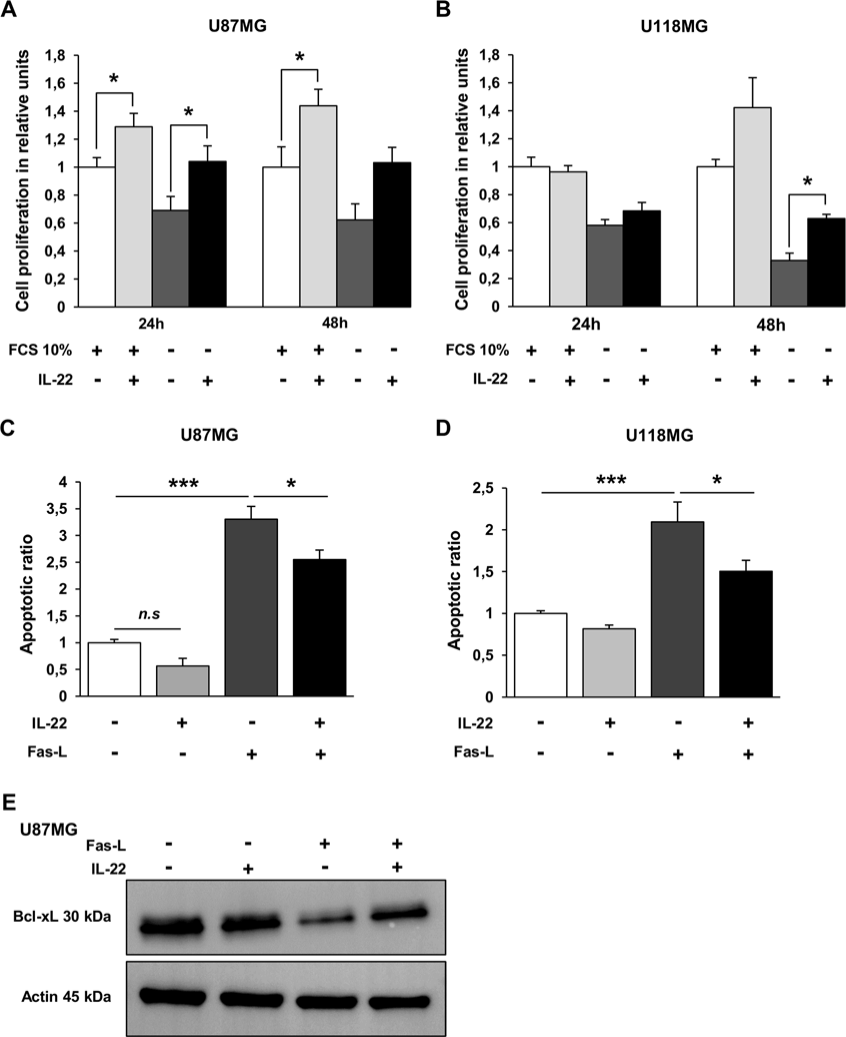
Exogenous IL-22 promotes cell survival of GBM cell lines. U87MG (A) and U118MG (B) cell lines were incubated for 24 to 48 h in basal culture medium (FCS 10%, +) and in serum-free medium (FCS 10%,-) in the presence of exogenous IL-22 (+) or without IL-22 (-). Cell proliferation was determined using BrdU cell proliferation assay. The data are represented as histograms of proliferating cells in relative units ± SEM of three independent experiments. *, a value of *p* < 0.05; when compared with respective control without exogenous IL-22. (C, D) Apoptotic ratios of soluble nucleosomes were detected by ELISA cell death for U87MG (C) and U118MG (D) cell lines induced after 48 h of incubation with Fas activation alone (Fas-L, +) or in combination with 20 ng/mL of recombinant IL-22 (IL-22, +). Histograms mean ratio of apoptotic cells ± SEM of three independent experiments. *, *p* < 0.05; ***, *p* < 0.001; when compared with respective control. (E) The antiapoptotic factor Bcl-xL expression was assessed by western blotting (in reference to actin) in total cellular protein extracted from cells treated or not with Fas-L, with or without recombinant IL-22 for 48 h.

Since IL-22R complex was expressed by GBM cells, we further searched for biological functions of IL-22. We thus performed proliferation assays with exogenous IL-22 either in FCS-free or in 10% FCS-containing cultures. A 24 h and 48 h incubation of U87MG cells with exogenous IL-22 induced cell proliferation assessed by BrdU assays, whatever the cell culture conditions (Fig. 2A). Proliferation is observed only at 48 h for U118MG cells, under the serum-free culture condition (Fig. 2B). To further evaluate the role of exogenous IL-22 on GBM cell survival, apoptosis was evaluated by measuring soluble nucleosome cytoplasmic levels in cultures in the presence or not of the Fas ligand-inducing apoptosis (Super Fas Ligand), as previously reported [34]. In the presence of the Fas ligand, IL-22 significantly decreases the apoptotic ratio of U87MG (Fig. 2C) and U118MG cell lines (Fig. 2D), whereas no significant effect was observed on cells cultured in medium alone (Fig. 2C and 2D), suggesting that IL-22 protects GBM cells from Fas ligand-induced apoptosis. Previous studies revealed the activation of the antiapoptotic protein Bcl-xL by IL-22 in lung cancer [20], colon cancer [22] and hepatocarcinoma [26] cells. We thus examined the relationship between Bcl-xL protein and IL-22 in U87MG cells. We showed that Bcl-xL protein is downregulated by Fas ligand and that IL-22 was able to rescue this effect (Fig. 2E), in accordance with the above-mentioned results obtained by the ELISA cell death assays.

### Interleukin-22 triggers phosphorylation of STAT3 in GBM cells

**Fig 3:**
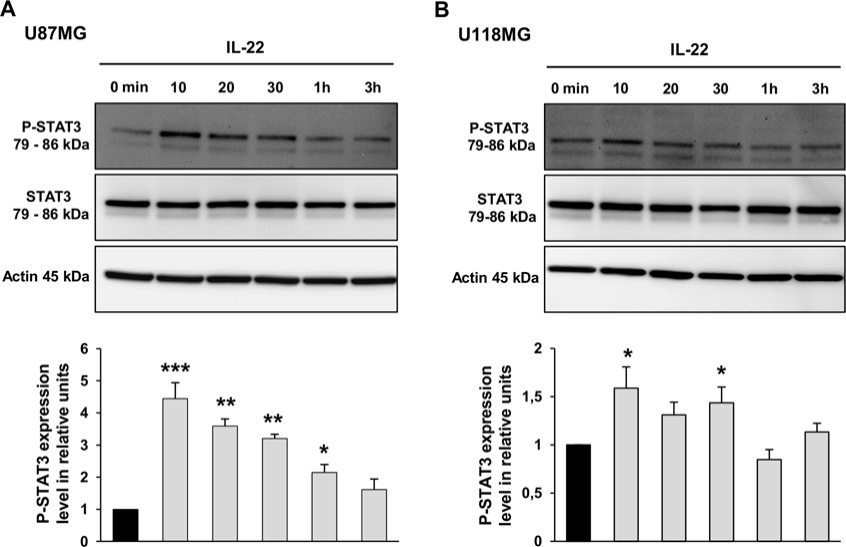
IL-22 activates the STAT3 signaling pathway in GBM cell lines. (A, B) The ability of IL-22 to activate signaling pathway in GBM cells was assessed using antibodies specific to STAT3 and Phospho-STAT3 (P-STAT3). U87MG (A) and U118MG (B) cells were stimulated with IL-22 and harvested at indicated times. Thirty mg of protein lysates was analyzed for P-STAT3 (Tyr705) and total STAT3 by western blot analysis. The density of each P-STAT3 band was corrected for variance in loading, using the density of the corresponding total STAT3. The expression level was evaluated as the ratio of phosphorylated STAT3 protein densities between control (0 min) and treated cells. Histograms are means ± SEM of three independent experiments. *, *p* < 0.05; **, *p* < 0.01; ***, *p* < 0.001; when compared with control.

**Fig 4:**
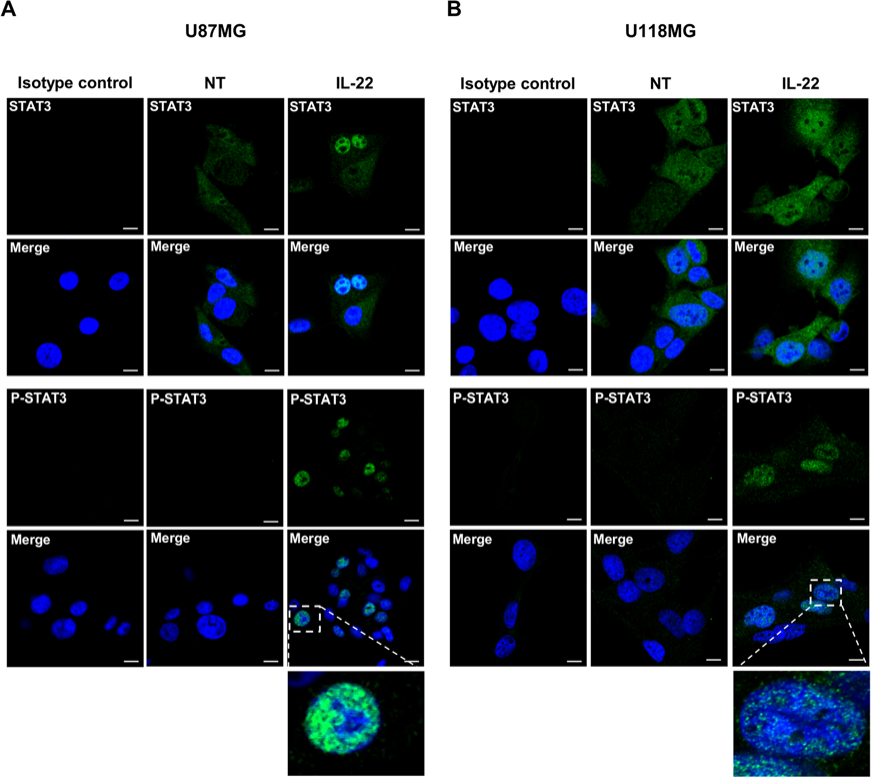
IL-22 induces STAT3 nuclear translocation and P-STAT3 nuclear accumulation in GBM cells. (A, B) Immunofluorescence analysis of STAT3 and P-STAT3 in U87MG (A) and U118MG (B) cells that were non-treated (NT) or treated with IL-22 for 30 minutes (IL-22). After the treatment, cells were fixed and stained with anti-STAT3 mouse mAb and anti-P-STAT3 rabbit mAb followed by Alexa fluor-conjugated fluorescent secondary antibody. Nuclei were counter stained with the blue-fluorescent DNA stain DAPI to point out nuclear localization of STAT3. Scale bars, 10μm.

To determine the signal transduction pathway induced by IL-22R activation, we searched for STAT3 phosphorylation in the two GBM cell lines following IL-22 stimulation. The treatment of U87MG by IL-22 induced STAT3 phosphorylation at Tyr-705, and the maximum phosphorylation levels were detected at 10 min (4.4-fold increase) and remained detected up to 3 h (1.6-fold increase) (Fig. 3A). Although weaker, IL-22 treatment of U118MG cells also increase the phosphorylation of STAT3 at 10 min (1.6-fold increase), 20 min (1.3-fold increase) and 30 min (1.4-fold increase) (Fig. 3B). Immunofluorescence studies on GBM cells further confirmed the IL-22-induced STAT3 phosphorylation, and showed its nuclear localization in both studied GBM cell lines after 10 min (data not shown) and 30 min of treatment (Fig. 4A and 4B), demonstrating the STAT3 nuclear translocation in the presence of IL-22 (Fig. 4A and 4B).

### Involvement of Akt and ERK1/2 pathways in IL-22-mediated cell survival in GBM cells

**Fig 5:**
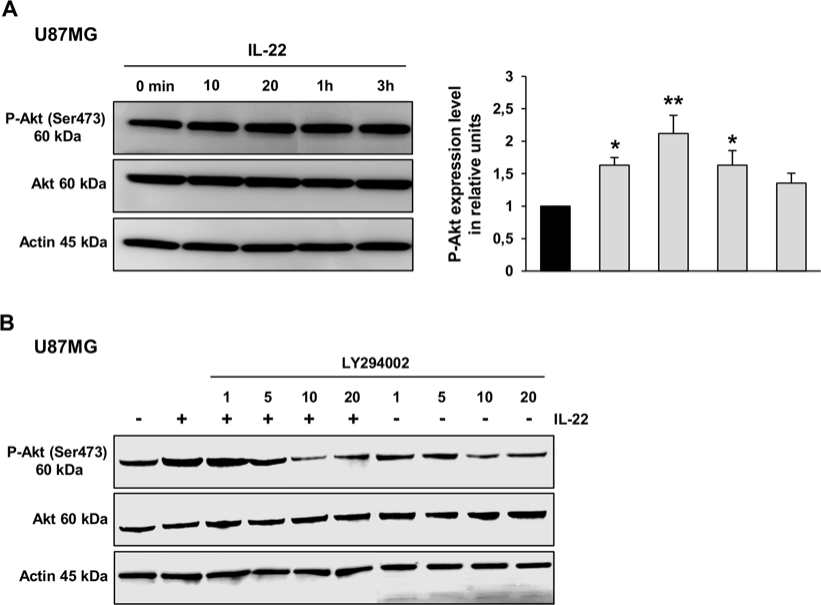
IL-22 enhances Akt phosphorylation in GBM cell lines. (A) The expression of phosphorylated Akt and the total amount of Akt were analyzed by western blotting for U87MG along a 3 h treatment with recombinant IL-22. Thirty mg of protein lysates was analyzed for P-Akt (Ser473) and total Akt by western blot analysis. The density of each P-Akt band was corrected for variance in loading, using the density of the corresponding total Akt. The expression level was evaluated as the ratio of phosphorylated Akt protein densities between control (0 min) and treated cells. A representative results of three independent experiments. *, *p* < 0.05; **, *p* < 0.01; when compared with control. (B) Western blot analysis of cellular protein extracted from U87MG cells pretreated with increasing concentrations of LY294002 for 2 h, then treated or not with recombinant IL-22 for 20 min. Thirty mg of protein lysates was analyzed for P-Akt (Ser473) and total Akt. Actin was used as a loading protein control.

IL-22 has also been shown to be involved in the activation of PI3Kinase-Akt pathway [8, 15, 16]. As shown for the U87MG cell line, the expression of phospho-Akt (Ser-473) was enhanced after 10 min of IL-22 treatment (Fig. 5A). IL-22 treatment of U118MG cells appears to be effective to increase the phosphorylation of Akt at 30 min (data not shown).

**Fig 6:**
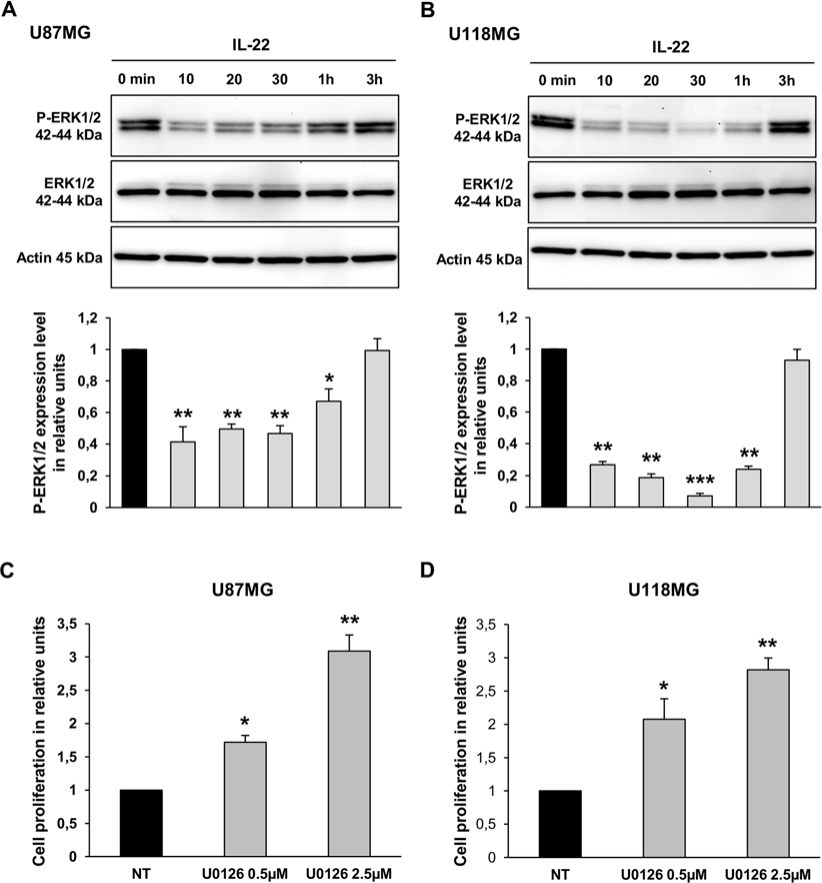
IL-22 reduces ERK1/2 phosphorylation in GBM cell lines. (A, B) The expression of P-ERK1/2 and the total amount of ERK1/2 were analyzed by western blotting in total cellular protein extracted from U87MG (A) and U118MG (B) cells treated with IL-22 for the indicated times. Thirty mg of protein lysates was analyzed for P-ERK1/2 and total ERK1/2. The density of each P-ERK1/2 band was corrected for variance in loading, using the density of the corresponding total ERK1/2. The expression level was evaluated as the ratio of phosphorylated ERK1/2 protein densities between control (0 min) and treated cells. A representative results of three independent experiments. *, *p* < 0.05; **, *p* < 0.01; ***, *p* < 0.001; when compared with control. (C, D) Effect of U0126 on proliferation of GBM cells. BrdU cell proliferation assays of U87MG (C) and U118MG (D) cells treated for 24 h in serum-free medium with vehicle (non-treated; NT) or with 0.5 and 5 μM of U0126. The data are represented as histograms of proliferating cells in relative units. Error bars indicate ± SEM. *, *p* < 0.05; **, *p* < 0.01.

IL-22-induced Akt phosphorylation was inhibited by pretreatment of U87MG cells with increasing concentrations of the PI3Kinase inhibitor (LY294002), confirming that this effect is indeed mediated by the PI3Kinase pathway (Fig. 5B). On the other hand, IL-22 treatment inhibited the phosphorylation of ERK1/2 in the U87MG (Fig. 6A) and the U118MG (Fig. 6B) cell lines. The decreased level of ERK1/2 phosphorylation was detected from 10 min of IL-22 treatment (1.6-fold decrease for U87MG and 1.7-fold decrease for U118MG) and remained lower than the control up to 1 h of treatment for both cell lines. Given that inactivation of ERK1/2 is not often correlated with cell proliferation in GBM cells, we examined the effect of the ERK chemical inhibitor (U0126) on GBM cell lines to see whether it could mimic the effect of IL-22 stimulation. Accordingly, 0.5 and 2.5 μM of U0126 significantly increased proliferation of both GBM cell lines (Fig. 6C and 6D). Altogether, these data suggest that IL-22 enhances GBM cell proliferation and cell survival *via* inactivation of ERK1/2 and activation of STAT3 and PI3K-Akt phosphorylation.

### Interleukin-22 receptor complex is expressed in GBM-initiating cells

**Fig 7:**
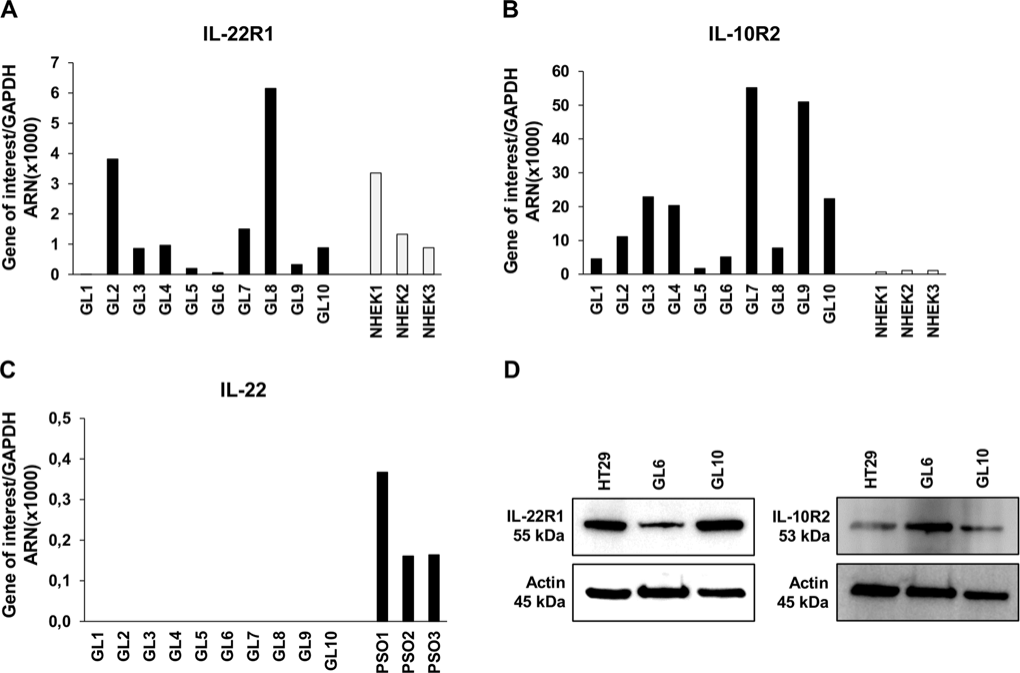
IL-22 receptors are expressed in human GBM tumors. (A-C) Quantitative RT-PCR analysis of IL-22R (IL-22R1, IL-10R2) and IL-22 in total RNA extracted from 10 GBM-initiating cells established from GBM tumors (GL). Three independent NHEK cultures were used as a positive control for IL-22R1 (A) and IL-10R2 (B) mRNA expression and three human psoriatic skin biopsies (PSO) were used as a positive control for IL-22 (C). Target gene expression was normalized to the housekeeping GAPDH mRNA. (D) Detection of IL-22R1 and IL-10R2 proteins assessed by western blot analysis from two GBM-initiating cells. Positive control was HT29 cell line. Actin was used as a loading protein control.

We further analyzed IL-22R expression in 10 GBM-initiating cells established from GBM tumors [33, 35]. Interestingly, mRNA expression of IL-22R1 and IL-10R2 was detected at various levels in all the samples, with prominent expression of the ubiquitous receptor component IL-10R2 (Fig. 7A and 7B). We used NHEK as positive controls, and showed expression levels higher for IL-10R2 but at similar levels for IL-22R1 in glioblastoma primary cells. Otherwise, IL-22 mRNA was not detected in all studied human GBM-initiating cells (Fig. 7C), while they were detected in psoriasis skin samples used as a positive controls [18]. In accordance with the transcriptomic results, protein expression of both IL-22R subunits was confirmed by western blot analysis in total cellular protein extracted from GL6 and GL10 GBM-initiating cells (Fig. 7D), confirming the results obtained with U87MG and U118MG cell lines.

## Discussion

The first reports on the involvement of IL-22 in physiopathology were in chronic inflammatory diseases, as psoriasis or colitis, since IL-22 contributes to tissue inflammation. A functional role of IL-22 in carcinogenesis has been therefore reported in lung, colorectal, gastric, pancreatic and hepatocarcinomas [1]. In the present study, we show the expression of the IL-22 receptor subunits IL-22R1 and IL-10R2 in GBM cell lines and in 10 GBM-initiating cells established from patients undergoing surgery for malignant GBM (WHO grade IV). All the 10 studied cell lines expressed both chains, with large variation of expression levels. Different studies have shown that IL-22 expression is associated with pro-survival and proliferation in a number of human cancer cells. In lung cancer, IL-22 induces cell proliferation and prevents from chemotherapy [20] and IFN-Ɣ [36]-induced apoptosis. In hepatocellular carcinoma, IL-22 induces cell survival and proliferation [13, 26]. IL-22 also enhances tumor growth and metastasis in both colon cancer [22] and pancreatic adenocarcinoma [24, 25]. In the current study, we showed that IL-22 protects GBM cells from Fas ligand-induced apoptosis and promotes cell proliferation. In agreement, IL-22 was able to increase the expression of the antiapoptotic protein Bcl-xL in U87MG cells treated with Fas ligand. We can hypothesize that the enhancement of cell proliferation is linked to the inhibition of apoptosis, allowing the proliferation of rescued cells. IL-22 has been also reported to involve different signaling pathways including STAT3, ERK, and Akt in cancerous cells [1], depending of the cell type. In liver, gastric or breast cells, IL-22 enhances tumor growth and progression by activating STAT3, followed by ERK1/2 [13, 21, 37] and/or Akt phosphorylation [8, 16]. However, in EMT6 breast cancer cells, IL-22 induces STAT3 phosphorylation, but reduced ERK1/2 and Akt phosphorylation [38]. In renal carcinoma cells, IL-22 suppresses cell growth in a dose dependent manner and inhibits the growth of tumor xenografts; these effects were in part mediated through regulation of STAT1 and ERK1/2 signaling pathways [39]. Despite these conflicting results, inhibition of ERK1/2 phosphorylation has been suggested as a treatment approach in breast cancer cells [40], lung cancer cells [41] and GBM cells. For example, it was reported that miltefosine-induced GBM cell apoptosis is dependent on ERK1/2 activation [42]. Similarly, allicin-induced apoptosis is regulated by MAPK/ERK-dependent pathway [43]. In our hands, IL-22 treatment promotes GBM cell proliferation and protects them from apoptosis *via* the phosphorylation of STAT3, Akt and inactivation of ERK1/2. Using confocal microscopy, we confirmed IL-22-induces P-STAT3 nuclear accumulation and STAT3 nuclear translocation, as recently described in oral squamous cell carcinoma [44]. The differential response of GBM cell lines to IL-22 stimulation could be explained by the higher expression of IL-22R in U87MG cell line. Otherwise, deregulated expression and/or mutations of PTEN (phosphatase and tensin homolog) and p53 have been described to act synergistically as activator of STAT3 signaling in tumors outside the brain, including breast cancer [45]. For this study we used U87MG (PTEN-mutant; p53-wt) and U118MG (PTEN-mutant; p53-mutant) cell lines, both classified as grade IV glioblastoma. We suggest that the reduced phosphorylation level of STAT3 induced by IL-22 in U118MG cells compared to U87MG could be explained by a higher constitutively activated STAT3 in PTEN/p53-comutant U118MG cells.

In many cancer cells, autocrine production of cytokines is essential for cell survival and proliferation. Recently, this autocrine mechanism was demonstrated in human lung cancer cells secreting IL-22- [20] and IL-6- [46] induced STAT3 phosphorylation, thus contributing to the pathogenesis of lung adenocarcinoma and formation of pleural effusion. An autocrine production of IL-4 and IL-10 has also been reported in thyroid carcinoma cells, promoting resistance to Fas-induced apoptosis through the activation of JAK/STAT pathways [47]. By contrast, GBM cells did not produce IL-22, whereas they express a functional IL-22 receptor, suggesting that IL-22 could be provided by microenvironmental cells. Recently, Th17 cells invasion was reported in experimental mouse model of malignant glioma as well as in human glioma [29]. In this context, it is tempting to speculate that the functional effect of IL-22 in GBM could be induced by neighboring immune cells such as Th17 cells. Additional studies will be helpful to confirm that Th17 cells are possible source of IL-22 in human GBM. Nowadays, it is known that GBM has a characteristic cytokine expression pattern, and dysregulations of normal cytokine-mediated cell proliferation have been implicated in gliomagenesis [48]. Amongst them, IL-6 and IL-10 share signaling pathways with IL-22. IL-6 is secreted by human GBM cell lines such as U87MG, promoting their invasion [49]. IL-6 expression was positively associated to the aggressiveness, histopathological grade and poor overall survival of patients with GBM [50]. IL-10 was described to be produced by primary cultured glioma cells as well as glioma cancer stem cells [51]. IL-10 inhibits human T cell proliferation by downregulation of MHC class II expression, inhibiting the antigen-presenting capacity of monocytes and subsequently promotes glioma tumor growth [48, 52].

The common feature of these cytokines involved in glioblastoma development is their implication in inflammatory response, underlying the fundamental implication of tumor microenvironment. Amongst the complex cytokine network involved in tumor progression, we suggest that immunocompetent cells can interact with tumor cells and secrete IL-22 to promote GBM tumor development. Indeed, previous studies reported that IL-22 expression is correlated with tumor invasion and poor overall survival in other types of human cancers [21, 26, 27]. Collectively, our results suggest that IL-22 is a new candidate able to play a role in GBM growth and progression. Thorough additional studies will be necessary to consider the IL-22/IL-22R axis as a novel therapeutic target for GBM patients.
